# Investigating the effect of poly (ɛ-caprolactone) nanofibers scaffolds with random, unidirectionally, and radially aligned morphologies on the Fibroblast cell’s attachment and growth behavior

**DOI:** 10.55730/1300-0527.3516

**Published:** 2022-11-08

**Authors:** Saeed NEZARI, Mohammadamin SARLI, Ahmad HIVECHI, S. Hajir BAHRAMI, Peiman B. MILAN, Noorahmad LATIFI, Fatemeh LATIFI, Tayyeb GHADIMI, S. Mohammad Amin HARAMSHAHI, Soheila NADERI GHARAHGHESHLAGH

**Affiliations:** 1 Department of Textile Engineering, Amirkabir University of Technology, Tehran, Iran; 2 Department of Plastic and Reconstructive Surgery, Hazrat Fatemeh Hospital, Iran University of Medical Sciences, Tehran, Iran; 3 Department of Textile Engineering, Ege University, İzmir, Turkey; 4 Cellular and Molecular Research Center, Iran University of Medical Sciences, Tehran, Iran; 5 Department of Tissue Engineering & Regenerative Medicine, Faculty of Advanced Technologies in Medicine, Iran University of Medical Sciences, Tehran, Iran; 6 Department of Oral and Maxillofacial Surgery, School of Dentistry, Shahid Beheshti University of Medical Sciences, Tehran, Iran

**Keywords:** Nanofiber, morphology, tissue engineering, biomaterials

## Abstract

In the last decade, significant progress in tissue engineering, repairing, and replacing organs has been achieved. The design and production of scaffolds for tissue engineering are one of the main areas which have attracted the researcher’s interest. In this regard, electrospinning is one of the most popular methods of nanoscale scaffold similar to extracellular matrix production. This paper reports the fabrication of scaffolds consisting of radially aligned PCL nanofibers by utilizing a collector composed of a central point electrode and a peripheral ring electrode. The chemical and physical properties were compared using SEM, FTIR, XRD, and DSC experiments, as well as biological performance using the MTT method and cell morphology with nanofibers with random and unidirectionally morphology. Results of this study showed greater physical and biological properties for radially aligned nanofibers which make them an excellent candidate for wound healing applications due to the guided cell growth on this type of nanofiber.

## 1. Introduction

Wound dressings are tissue engineering scaffolds that assist the healing of wounds caused by burns, cuts, trauma, and diabetes [1]. These dressings are fabricated differently depending on the type of wound [2]. In order to find the best scaffold for each wound, various studies have investigated scaffolds with different physical or chemical properties [1,3]. Nanofiber scaffolds are frequently used as wound dressings due to their low cost of production, high specific surface area, processability, and a remarkable similarity to the extracellular matrix (ECM) of skin [4]. Nanofibers have been utilized to treat diabetic and burn wounds [5].

The nanofibrous structure can be tailored for the final application by altering the biopolymers and production process. The first thoroughly studied strategy is utilizing a biopolymeric combination for scaffold fabrication [5,6]. Natural biopolymers are biocompatible, biodegradable, and hydrophilic but exhibit poor mechanical properties and resistance to aqueous environments [7]. Synthetic polymers, in contrast, are produced more easily and have superior mechanical properties as well as resistance to aqueous media. Compared to natural biopolymers, they are less hydrophilic, biodegradable, and biocompatible. Therefore, it is advised to combine the aforementioned biopolymers so that their positive qualities balance out their respective negative characteristics [8]. For instance, Sharahi et al. produced scaffolds containing lignocellulosic nanoparticles synthesized from walnut shells with PCL and gelatin for tissue engineering applications [9]. In another study, Ranjbar et al. investigated the properties of keratine/polyvinyl alcohol and PCL for skin regeneration [10]. Hivechi et al. examined the effect of cellulose nanocrystals in PCL/gelatin nanofibers for wound healing applications [11].

Numerous techniques have been reported so far for producing nanofibers, the majority of which have produced nanofibers with random morphology. The nanofiber scaffold surface morphology can also be modified to adjust its final properties. Recently, novel techniques have been developed to give nanofibers a specific alignment. This alignment can be in one direction or radially aligned. Recent publications claim that physical characteristics like morphology and surface roughness impact biological responses and cell growth [12,13]. For example, Cooper et al. investigated the cellular compatibility of aligned PCL-chitosan nanofibers for nerve tissue engineering and revealed that aligned nanofibers effectively promote cell growth compared to random nanofibers [14]. Some studies, including the study of Refaaq et al., have shown that changing surface topography can influence cell migration [15]. Many studies have shown that surface topography, such as proliferation and stem cell differentiation, may affect cell behavior. Additionally, nearly all cell behaviors and properties may be affected by the alignment and even diameter of nanofibers. Xie et al. demonstrated that aligned poly (L-lactic acid) nanofibers outperformed aligned microfibers and random fibers in terms of proliferation and osteogenic differentiation in bone marrow mesenchymal stem cells [16]. In a different study, Xu et al. demonstrated a synergistic effect between chemical and biophysical signals from aligned nanofibers on the differentiation of human dermal fibroblasts and human umbilical vein endothelial cells, which led to the expression of extracellular matrix protein from dermal fibroblasts and angiogenic growth factors from endothelial cells. Furthermore, their study showed that not only these effects could be demonstrated in vitro but also cells that receive biophysical and chemical signals synchronously can enhance wound healing in vivo [17]. Studies indicated that fiber alignment leads to cell alignment and increases the expression of genes related to focal adhesion and cytoskeleton [18].

Recently developed nanofibers with radially aligned morphology are used in biomedical applications. With the assistance of a collector made up of a central point and periphery ring electrode, Xie et al. developed radially aligned PCL nanofibers for the first time. They reported that the cells grew from the outermost part of the nanofibrous scaffold to the center of this construct [19]. Shim et al. fabricated radially aligned fibrous scaffolds coated with polydopamine for guiding directional migration of human mesenchymal stem cells [20]. In another study, Kim et al. fabricated a 3D electrospun scaffold suitable for treating ocular tissues injury due to the hemispherical shape and radially aligned nanofibers which can guide the direction of the main collagen and cellular actin filament in the extracellular matrix [21].

According to the literature review, various studies have been conducted on nanofiber fabrication with unidirectionally and radially aligned nanofibers. However, no comparative study has been reported on fibroblast cell biocompatibility in any of these studies. Therefore, the current study’s main objective was to investigate previously unreported effects of different morphology on the growth of fibroblast cells in the same experimental environment. The current manuscript involves the fabrication of nanofibers with a random, unidirectional, and radially aligned morphology. Then their physical characteristics are investigated using scanning electron microscopy (SEM), fourier transform infrared spectroscopy (FTIR), differential scanning calorimetry (DSC), and X-ray diffraction (XRD). Moreover, their biological properties, such as biocompatibility behavior, are studied. We hypothesize that radially aligned nanofibers will have the same chemical composition but different physical properties. In addition, we believe that the nanofiber alignment will influence faster fibroblast cell growth and a shorter healing process.

## 2. Materials and methods

### 2.1. Materials

All chemicals in this study were analytical grade. Polycaprolactone (PCL) (MW= 80000 Da), penicillin-streptomycin solution, trypsin, and methylthiazolyldiphenyl-tetrazolium bromide (MTT) were purchased from Aldrich. Phosphate-buffered saline (PBS) tablet, acetic acid, dimethyl sulfoxide (DMSO), and potassium bromide (KBr) were provided by Merck. The fetal bovine serum (FBS) was bought from Bioidea.

### 2.2. Nanofiber fabrication

In this research, nanofibers with random, unidirectionally, and radially aligned morphology are produced. The electrospinning process for the mentioned nanofibers used the same polymer solution. PCL granules were dissolved in 90% acetic acid at a concentration of 15% (w/v) and mixed for 6 h to get a homogeneous polymer solution [22]. For random morphology, the polymer solution was transferred into a 10 ml syringe (19 G) and electrospun at 17 kV, 0.9 mL/h feeding rate, and 16 cm needle tip to collector distance. Also, unidirectional nanofibers were fabricated using the same electrospinning parameters on a rotating collector (2000 rpm). Special collector designs that contain a central point and a peripheral ring electrode are used to produce radially aligned nanofibers. This type of nanofiber was electrospun at 15 kV, 0.5 mL/h feeding rate, and a needle tip to collector distance of 10 cm. The relative humidity and temperature were respectively 50% ± 5% and 25 ± 1 °C during the electrospinning procedures.

### 2.3. Nanofiber characterization

#### 2.3.1. Scanning electron microscopy (SEM)

First, a small piece of each sample was mounted using carbon glue on an SEM sample holder. Then, Bal-tec equipment was used to sputter a thin gold layer onto nanofibrous samples. The morphology of the electrospun nanofibers was analyzed using a Seron AIS2100 SEM at 5 kV accelerating voltage and ≈12 cm working distance. The average diameter of nanofibers was determined using Image J image analysis software.

#### 2.3.2. Fourier transform infrared spectroscopy (FTIR)

The samples were ground into small pieces using a ceramic porcelain pestle and mortar, then mixed with KBr salt and pressed into a tablet. These tablets were then mounted in a NEXUS 670 (Thermo Nicolet Co. USA) FT-IR spectrophotometer. The FTIR spectrum was then recorded in transmittance mode from 400–4000 cm^−1^ wavenumber, with 40 scans conducted for each sample.

#### 2.3.3. Differential scanning calorimetry (DSC)

About 5 mg of each sample was placed in a sealed DSC pan and transferred into a DSC 2010 TA instrument. The samples were heated at a rate of 10 °C/min from 0 to 90 °C. They were then kept at 90 °C for 10 min to remove the polymer’s thermal history. Finally, they were cooled to 0 °C at a rate of 10 °C/min.

#### 2.3.4. X-ray diffraction (XRD)

X-ray diffraction measurement was used to investigate the crystalline structure of electrospun nanofibers with different morphologies. First electrospun meshes were detached from the collector and placed in an X-ray sample holder, and the spectrum was recorded using Inel Equinox 3000 XRD equipment. The X-ray diffraction pattern was recorded from 5° to 80° in reflectance mode using a Cu K_α_ beam (λ of 1.54 Å) at a step width of 0.25° min^−1^.

### 2.4. Nanofiber biological properties

#### 2.4.1. MTT assay

L929 cell line was used for the MTT assay. After being sterilized with UV light for 2 h and submerged for 30 min in 70% (v/v) ethanol, nanofiber mats were placed in 96 well plates. Then 100 μL of 3 × 10^3^ cells maintained in cell culture media (DMEM, 10% FBS, 100 U penicillin/streptomycin) were seeded in each well. The samples were incubated at 37 °C, 5% CO_2_, and 95% relative humidity, and the culture medium was changed every 48 h after incubation for 1, 3, and 7 days, the culture medium was removed, and 100 μL MTT solution (0.5 mg/mL) was added to each well and incubated at 37 °C for 4 h. The MTT dye reacts with cells and produces formazan precipitates. Then 100 μL DMSO was added to each well after removing the MTT solution. Note that the control sample consists of wells without nanofibers. Optical density was measured using a spectrophotometer. The cell viability was then calculated using the following equation.


(1) $$\text{Cell viability }(\%)=\frac{{\text{OD}}_{\text{d}}-{\text{OD}}_{\text{c}}}{{\text{OD}}_{\text{c}}}\times 100$$

Where OD_s_ and OD_c_ are the respectively average optical density for the examined substrate and control sample. This experiment was replicated three times.

#### 2.4.2. Cell culture

The morphology of the cultured cells was examined using scanning electron microscopy (SEM). Electrospun mats were placed in 24 well cell culture plate after sterilization. Then 1 × 10^4^ fibroblast cells were seeded on each mat. Afterward, Plate was incubated at 37° C, with 95% relative humidity and 5% CO_2_ for 24 h. Then culture medium was removed, and cells were fixed using 2.5% (v/v) glutaraldehyde aqueous solution at 4 °C overnight. Finally, glutaraldehyde was discarded, and samples were washed using distilled water to remove unreacted chemicals or salt precipitates. Then samples were dried in a freeze drier at −80 °C overnight.

## 3. Results and discussion

### 3.1. Nanofibrous scaffold characterization

**Figure 1:**
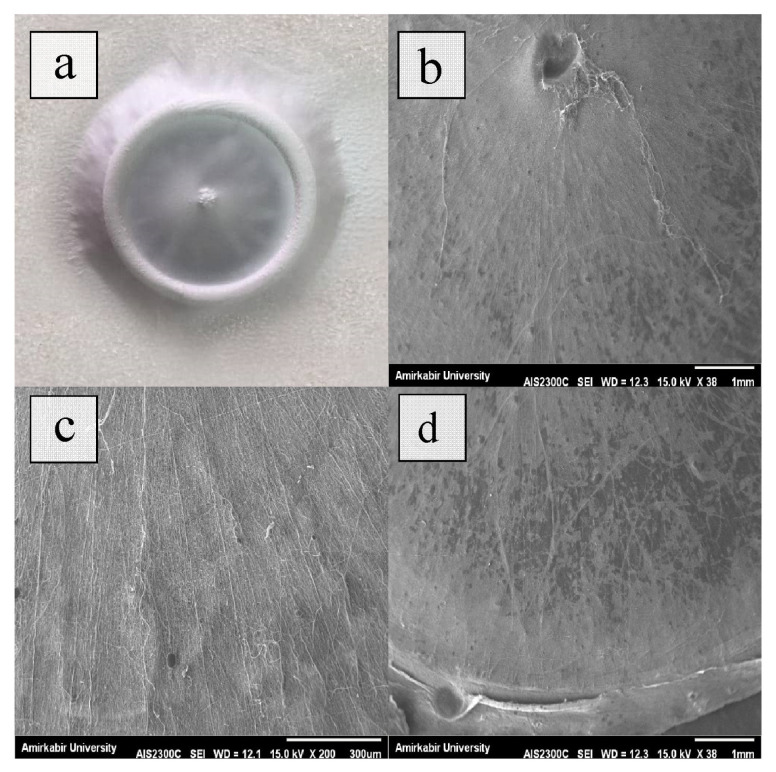
Low magnification of produced radially aligned nanofibers on the collector.

Radially aligned nanofibers at various sites were recorded in low magnification SEM pictures (Figure 1). These results confirm that radially aligned nanofibers were successfully fabricated. The polymer solution is first stretched to the collector’s central point due to the applied high voltage. The produced fiber is then stretched again due to the collector design, with one end placed on the center point and the other on the circular electrode. Because of this, most of the polymer is centered on the center point.

**Figure 2:**
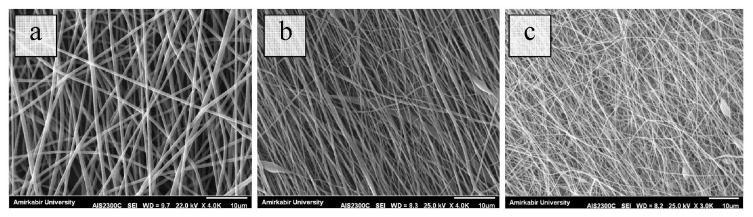
SEM images of (a) random, (b) unidirectionally, and (c) radially aligned nanofibers.

Figure 2 shows micrographs of produced nanofibers oriented at random, unidirectionally, and radially aligned. The average diameters were respectively 240 ± 45 nm, 257 ± 65 nm, and 226 ± 64 nm. The statistical analysis revealed that there was no significant difference between the groups. However, radially aligned nanofibers exhibited the least diameter, whereas randomly and unidirectionally nanofibers displayed almost equal diameters. Radially aligned samples have smaller nanofiber diameters due to the extra tension applied during the electrospinning process.

**Figure 3:**
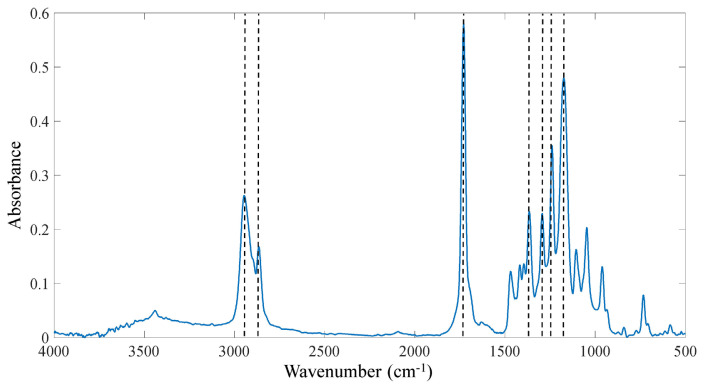
FTIR spectrum of PCL nanofiber.

The functional groups of produced samples were analyzed using FTIR spectroscopy, shown in Figure 3. The peaks observed at 2947 cm^−1^ (C-H stretch sp^3^ asymmetric), 2866 cm^−1^ (C-H stretch sp^3^ symmetric), 1730 cm^−1^ (C=O stretch ester), 1468 cm^−1^ (CH_2_ bending oop), 1365 cm^−1^ (CH_3_ bending oop), 1294 cm^−1^ (C-O and C-C stretch in crystalline phase), 1240 cm^−1^ (C-O-C stretch asymmetric) and 1174 cm^−1^ (C-O-C stretch symmetric) are confirming the molecular structure of the PCL polymer.

**Figure 4:**
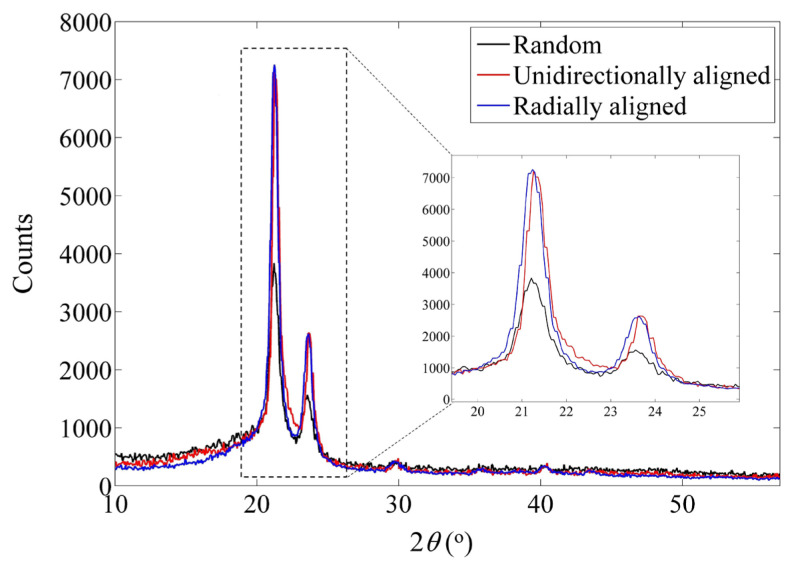
X-ray diffraction patterns of random, unidirectionally, and radially aligned PCL nanofibers.

Analyzing the crystalline structure of the produced nanofibers with different morphologies was examined by the X-ray diffractometer (Figure 4). There were minor variations (±0.3) in peaks because of differences in sampling or measurement procedure. According to the Miller plans (110) and (200), all samples showed similar patterns with two peaks at 21.2° and 23.6°. We could not evaluate the crystallinity using X-ray patterns since we could not access a perfectly crystalline PCL sample. Thus, we utilized a method reported by Hivechi et al., which uses the peak ratio (Equation 1) to compare the relative amounts of crystalline phase [23].


(2) $$\text{Peak ratio}=\frac{{\text{I}}_{2\theta =21.2}-{\text{I}}_{2\theta =22.7}}{{\text{I}}_{2\theta =22.7}}$$

The peak ratios for random, unidirectional, and radially aligned nanofibers were 4.29, 5.78, and 7.29, respectively. As a result, random nanofibers exhibit the lowest crystallinity, while the radially aligned samples show the highest crystalline phase. During the electrospinning process, the fibers are drawn due to the electrostatic forces. In the unidirectionally aligned sample, extra mechanical tension is applied to nanofibers due to collector rotational speed, resulting in a higher drawing rate and further polymer alignment, and a higher crystalline phase. In the radially aligned sample, the electrostatic force is applied to fibers in two stages, first from the nozzle to the collector and second from the central point to the outer circular electrode. Therefore they are drawn twice during the fiber fabrication. This has resulted in a higher possibility of crystalline phase formation. When the solvent evaporates for electrospinning, molecular chains fold locally along the fiber axis, forming a crystallite. Wang et al. reported that PCL chain folding occurred along the [110] and [010] growth planes in a PCL lamellar single crystal, as it does in other aliphatic polyesters, and the polymer chains were mostly aligned perpendicular to the crystal’s base plane [24]. In particular, electrospun PCL nanofibers possess the following crystalline structure: (1) The molecular chains and crystallites themselves are highly oriented along the fiber axis. (2) The nanofibrils in nanofibers are produced by crystallites aligned along the fiber axis. (3) A single nanofiber is composed of numerous nanofibrils.

**Figure 5:**
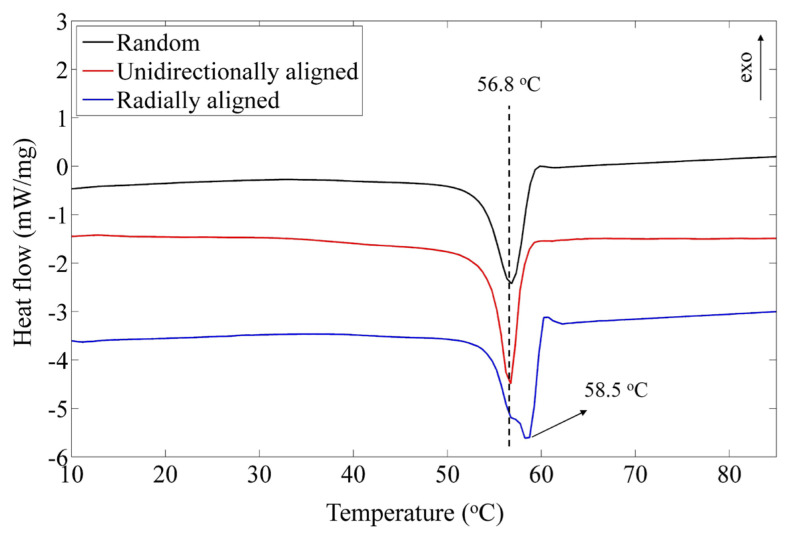
DSC curves of random, unidirectionally, and radially aligned PCL nanofibers.

Figure 5 illustrates how the thermal properties of the produced nanofibers were evaluated using DSC. The obtained data from these curves are given in Table. Random PCL nanofibers showed a melting point (T_m_) at 56.8 °C with melting enthalpy (H_m_) of 49.7 J/g. The crystalline volume can be calculated according to equation 2.


(3) $${\text{X}}_{\text{c}}(\%)=\frac{{\text{H}}_{\text{m}}}{{\text{H}}_{\text{m}}^{\text{o}}}\times 100$$

Where H_m_^o^ is the melting enthalpy of perfectly crystalline PCL, and X_c_ (%) is the volume percentage of the crystalline phase. Sheikholeslami Kandelousi et al. have reported that H_m_^o^ for PCL is 136 (J/g) [25]. The crystalline volume is calculated based on this value, and the results are reported in Table. On the other hand, the melting point is representative of crystal size. To put it another way, higher melting temperatures indicate a larger crystal size. According to the results, both the random and unidirectionally aligned nanofibers have the same crystalline volume. But the onset (T_mo_) and final (T_mf_) melting points were respectively 50.8 °C and 59.2 °C. As can be seen from this observation, the crystal sizes in this sample are more uniform than those at random. During nanofiber fabrication, mechanical tension could have caused this phenomenon. Compared to unidirectionally aligned samples and random samples, radially aligned samples displayed different thermal behavior. Based on melting enthalpy, 50% of the sample was shown to be composed of a crystalline phase. Due to the double electrostatic tension mentioned earlier, this has occurred. Moreover, we observed two melting points in this sample: the first at 56.8 °C and the second at 58.5 °C. We believe polymers are located on the center and outer circular electrodes that cause the first melting point. While the second melting point, stronger than the first, could be attributed to nanofibers between electrodes. Therefore, we can conclude that there are two crystal sizes in this sample: small and large.

### 3.2. Nanofiber biological properties

**Figure 6:**
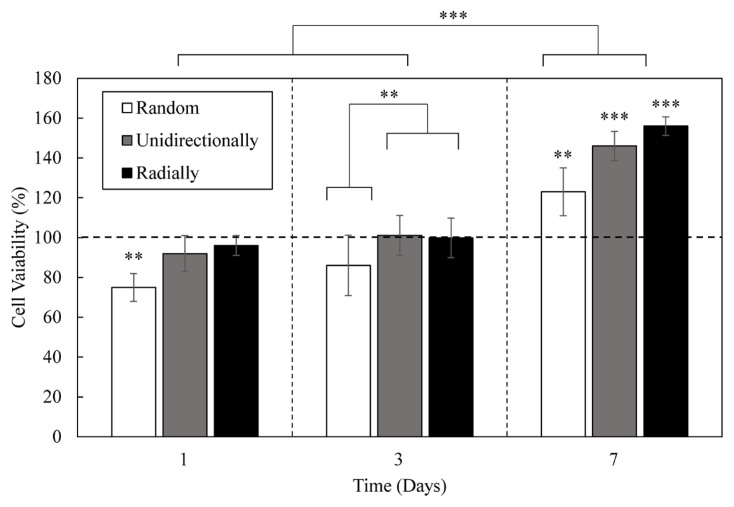
Cell viability results for random, unidirectionally, and radially aligned PCL nanofibers obtained from MTT assay. The horizontal dashed line shows the cell viability for the control sample. The significantly different groups from the control samples are illustrated on each bar with * (p < 0.05), ** (p < 0.01), and *** (p < 0.001). Samples and days with significant differences are demonstrated by *, **, and ***. The overall post hoc results for treated groups with different morphologies and cell culture time are illustrated on day three and top of the graph.

The biocompatibility of the nanofibers at different time intervals is tested using the MTT assay, and its results are reported in Figure 6. Earlier research articles have shown that toxic groups are considered to have a lower level of cell viability than 80%; otherwise, the sample will be considered biocompatible. According to the results, the biocompatibility was achieved for all samples except the random nanofibers on the first day of cell culture. At the beginning of the cell growth process, weak cell attachment to PCL nanofibers may have caused this phenomenon. This hypothesis can be confirmed by increased cell viability for random nanofibers after 3 and 7 days. Unidirectionally and radially aligned nanofibers showed superior cell compatibility compared to random nanofibers. According to the statistical analysis, both nanofibers with alignment showed significantly stronger cell viability. This observation confirms our initial hypothesis that alignment plays an essential role in cell growth and proliferation. Additionally, the number of cells increased significantly after seven days of cell culture compared to the first and third days. This finding confirms the successful cell growth over time, which enables the application of nanofiber scaffolds as a wound dressing.

**Figure 7:**
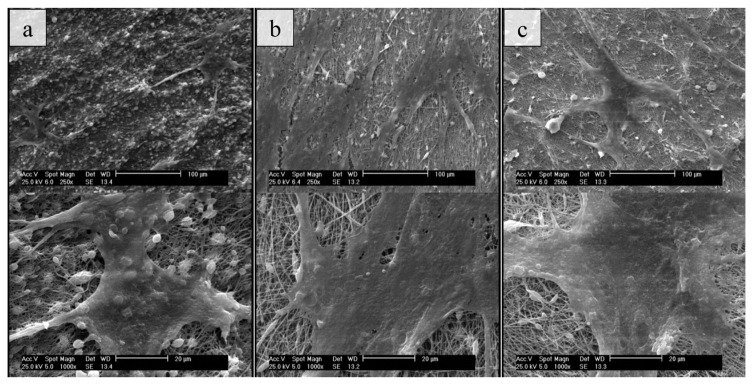
SEM images of cultured cells on (a) random, (b) unidirectionally, and (c) radially aligned PCL nanofibers.

The effect of nanofiber scaffold morphology on cell attachment was studied using SEM images of cultured cells on nanofiber samples (Figure 7). The results indicate that all nanofiber structures sufficiently attach to cells due to their nano dimensions and high specific surfaces. However, for random, unidirectionally, and radially aligned morphologies, their shape, and covered area exhibited significant differences. Random nanofibers had small numbers of cells, but aligned nanofibers had plenty in a similar area. These results were equivalent to MTT assay results that showed higher cell biocompatibility for aligned samples. The average area covered by each cell was respectively 38,991, 109,563, and 127,143 μm^2^ for random, unidirectionally, and radially aligned nanofibers. These findings were comparable to the MTT assay, which shows improved cell adhesion and growth on aligned nanofibers. Thus, the alignment of nanofibers can significantly affect the cell growth behavior.

## 4. Conclusion

In summary, we successfully produced PCL nanofibers with random, unidirectionally, and radially aligned morphologies. The chemical characterization showed that there is no significant difference in their chemical structure. However, their physical properties, such as crystallinity and thermal behavior, were influenced by the electrospinning technique. Results from DSC and XRD studies indicated that radially aligned nanofiber exhibits higher crystalline portion and thermal behavior, which is essential for biomedical applications. Moreover, biological studies also showed better performance of aligned nanofibers that can promote cell attachment and growth. Therefore results of this study suggest aligned nanofibers for wound healing applications.

## Figures and Tables

**Table:** Melting point (T_m_), onset (T_mo_), and final (T_mf_) temperatures of melting process for random, unidirectionally, and radially aligned PCL nanofibers. Based on DSC curves, melting enthalpy (H_m_) was used to calculate the percentage of the crystalline phase.

Sample	T_mo_ (°C)	T_m_ (°C)	T_mf_ (°C)	H_m_ (J/g)	X_c_ (%)
Random	50.3	56.8	59.8	49.7	36.5
Unidirectionally aligned	50.8	56.7	59.2	48.2	35.4
Radially aligned	51.7	56.8, 58.5	60.2	59.6	43.8
